# Rotationplasty performed in adults versus minors: a comparative study of long-term quality of life, functional and biomechanical outcomes

**DOI:** 10.1016/j.jbo.2025.100732

**Published:** 2025-11-26

**Authors:** Gitte G.J. Krebbekx, N.F.J. Waterval, M.A. Brehm, M.J.C. Duivenvoorden, I.N. Sierevelt, J.A.M. Bramer, G.M.M.J. Kerkhoffs, F.G.M. Verspoor

**Affiliations:** a Department of Orthopedic Surgery and Sports Medicine, Amsterdam UMC, Amsterdam Movement Sciences, University of Amsterdam, Meibergdreef 9, Amsterdam, the Netherlands; b Department of Rehabilitation Medicine, Amsterdam University Medical Center, Meibergdreef 9, Amsterdam, the Netherlands; c Department of Orthopaedic Surgery, Xpert Clinics, Orthopedic Department, Laarderhoogtweg 12, Amsterdam, the Netherlands; d Department of Orthopaedic Surgery, Spaarnegasthuis, Orthopedic Department Spaarnepoort 1, Hoofddorp, the Netherlands; e Amsterdam Movement Sciences, Rehabilitation & Development, Amsterdam, the Netherlands; f Cancer Center Amsterdam, Amsterdam, the Netherlands

**Keywords:** Tumor, Sarcoma, Rotationplasty, Adults

## Abstract

**Background:**

Rotationplasty is a surgical technique primarily used in pediatric patients with malignant bone tumors around the knee. Its application in adults is rare, and outcomes are poorly defined. This study aimed to compare long-term QoL, function, and gait between patients treated with rotationplasty in childhood versus adulthood.

**Methods:**

This cross-sectional post-hoc predefined secondary analysis included 33 patients treated between 1980–2002 (9 adults, 24 minors). Assessments included the mental and physical component score of the Short Form-36 (SF-36, MCS/ PCS), overall satisfaction, radiographs (Kellgren-Lawrence) of the pseudo-knee, contralateral ankle, and hips, Toronto Extremity Salvage Score (TESS), Musculoskeletal Tumor Society Score (MSTS), energy cost of comfortable walking during a 6-minute walk test, and 3-Dimenional gait analysis. Group comparisons were performed across all outcomes. Additionally, a systematic literature search identified published adult cases.

**Results:**

People who underwent surgery during adulthood reported significantly higher physical QoL compared to those operated in childhood (MD 6.1, 95 % CI 0.0–12.3; *p* = 0.05). No significant differences were observed for the SF-36 MCS, TESS, MSTS, energy cost, sagittal-plane biomechanics, and radiographic OA prevalence. Adults exhibited a significantly shorter stride length (p < 0.01) and a longer double support phase (p = 0.01) compared to those treated in childhood. The literature review (22 studies, 51 patients) demonstrated overall favorable outcomes, though no objective gait data were reported.

**Conclusion:**

Adults who undergo rotationplasty can achieve long-term quality of life, functional, and gait outcomes at least comparable to those operated on in childhood. These findings suggest that rotationplasty may be a viable reconstructive option in adults.

## Highlights

• Rotationplasty performed in adulthood showed higher physical QoL scores than minors.
• No differences in mental QoL, gait, energy cost, or radiographic OA were found.
• Adults showed shorter stride length and longer double support phase.
• Literature showed good QoL and function in adult cases, despite complications.

## Graphical abstract

**Figure:**
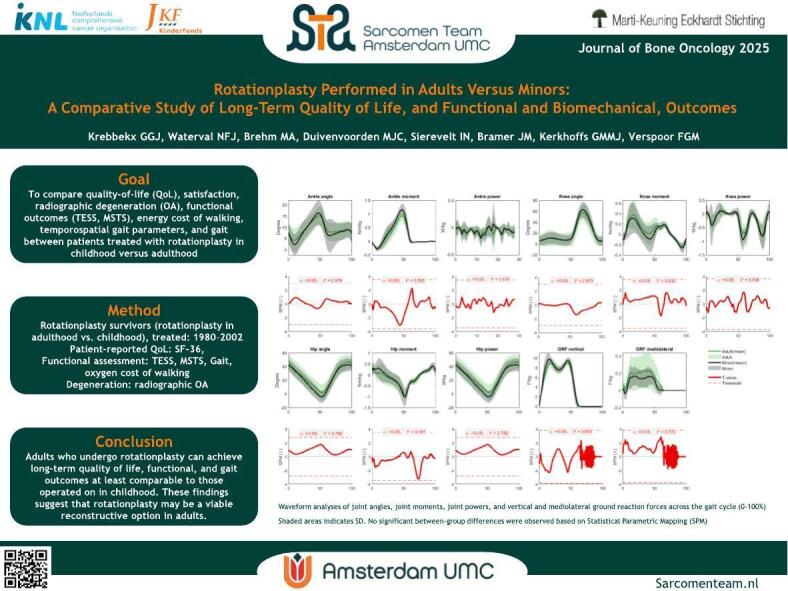

## 1 Introduction

Rotationplasty, also known as the Borggreve-Van Nes procedure, is a surgical technique that involves the resection of a diseased segment around the knee, followed by a 180-degree rotation and reattachment of the distal limb to the proximal femur [1,2]. This procedure reorients the ankle to function as a pseudo-knee joint, enabling the use of a below-knee prosthesis. While rotationplasty primarily performed in pediatric patients with tumors around the knee, it has also been described for congenital deformities, severe trauma, failed limb-salvage procedures, and infections after arthroplasty, conditions more commonly encountered in adults [1,[3], [4], [5], [6], [7]].

Compared to above-knee amputation, rotationplasty has several advantages: it preserves limb length, provides more efficient gait mechanics, allows greater prosthetic control, and reduces the incidence of phantom limb pain [8,9]. Although rotationplasty is often perceived as a last resort option after limb salvage procedures, patient-reported outcomes have been shown to be comparable or even superior to those of other (reconstructive) strategies[[10], [11], [12], [13]]. In the context of growing emphasis on shared decision-making in oncological and reconstructive surgery, rotationplasty should therefore be regarded a legitimate reconstructive option alongside conventional approaches [14].

Despite its effectiveness in minors, the application of rotationplasty in adults remains uncommon. While the surgical objective, restoration of functional independence and reintegration into daily life, is the same, adults may face higher risks of complications such as vascular compromise, infection, and nonunion, which may necessitate reoperation [15,16]. In addition, the distinctive cosmetic appearance of the limb and altered biomechanics can impose psychological stress [17]. Although long-term studies in children and adolescents suggest that psychological adaptation improves over time [18,19], robust data in adults undergoing rotationplasty are currently lacking.

Current evidence in adults is limited to case reports [7,20,21] and small case series[22,23]. Although these collectively provide a substantial body of observations, the results are heterogeneous and sometimes contradictory. Some studies report adults achieving satisfactory functional outcomes, including return to work and recreational activities [24,25], whereas others reported reduced mobility, increased reliance on walking aids, and less favorable gait biomechanics [17,26]. These differences may be attributed to age-related factors, such as reduced bone remodeling capacity, lower adaptability, and the absence of growth potential, which generally favor younger patients [7,27,28].

Given the scarcity of comprehensive data and the absence of long-term follow-up studies, there is a clear need to better define outcomes and assess whether rotationplasty can be recommended as a durable reconstructive option for adults. Because the existing reports are scattered and heterogeneous, these outcomes are difficult to interpret, and single case reports do not allow for evidence-based recommendations. A study including long-term outcomes combined with a systematic search to deliver the most complete overview to date is therefore warranted.

This study compared long-term outcomes of patients who underwent rotationplasty in adulthood with those who underwent the procedure during childhood or adolescence, focusing on quality of life, satisfaction, radiographic degeneration, functional outcomes, energy cost of walking, temporospatial gait parameters, and gait biomechanics. In addition, this study aimed to provide the most comprehensive overview to date of all currently available data on adult rotationplasty through a systematic search of the literature.

## 2 Methods

### 2.1 Study design and participants

This cross-sectional study, approved by the Amsterdam UMC ethics board (NL72453.018.20), is a predefined secondary analysis of a historical cohort of 70 patients who underwent rotationplasty between 1980 and 2002 at Amsterdam UMC and OLVG [29]. All procedures were Winkelmann type A1 resections for lesions of the distal femur or proximal tibia, performed by a single surgeon using a standardized technique [2]. The sciatic nerve was carefully preserved and mobilized, in most cases, a vascular arterial and venous anastomosis was created and in all cases fixation was achieved by plating and screwing the tibia onto the femur. Of these, 29 patients had died and six were lost to follow-up. Among the remaining 35 patients, two lived abroad. Ultimately, 33 participants were eligible and included for QoL questionnaires, 30 for OA imaging, and 29 for gait and energy-cost assessments, after signing the informed consent [29]. Participants were categorized according to their age at the time of surgery, with those younger than 18 years classified as minors and those 18 years and older as adults.

### 2.2 Data collection

Patients were invited to attend a single visit at Amsterdam UMC for a comprehensive clinical assessment, consisting of a standardized physical examination, acquisition of radiographic images, administration of functional outcome measures (MSTS and TESS), 3D gait analysis, and measurement of energy cost of walking. Patient-reported outcomes were collected during follow-up visits and through secure electronic questionnaires, administered within one week before the study visit to minimize recall bias. Demographic and clinical variables (sex, age at surgery, follow-up duration, tumor type, metastases, adjuvant therapy, complications, employment, and relationship status) were retrieved from medical records and patient self-report.

### 2.3 Outcome measures

#### 2.3.1 Quality-of-life

Health-related QoL was assessed using the Physical component score (PCS) and Mental component score (MCS) of the 36-item Short Form Health Survey (SF36). The PCS and MCS were calculated using standardized, population-based scoring algorithms [30]. A score of 50 represents the mean of the general population, with higher scores indicating better health status [31,32]. The SF-36 has been validated for use in healthy populations [33,34] and in in lower-extremity sarcoma patients [35].

Patients also rated their overall satisfaction with the likelihood of choosing the procedure again on a 0–100 visual analogue scale (VAS).

#### 2.3.2 Functioning

The patient-reported Toronto Extremity Salvage Score (TESS) was used to evaluate patient-reported functioning (score range: 0–100), which assesses physical disability across daily living, work, social, and sexual functioning. It is validated for Dutch sarcoma patients [[36], [37], [38]]. The Musculoskeletal Tumor Society Score (MSTS) was used to evaluate pain, function, emotional acceptance, use of supports, walking ability, and gait. Each item was scored from 0 to 5, yielding a total of 30. To ensure consistency, all MSTS assessments were administered and scored by a single physician [39,40]. The MSTS is a validated tool to assess limb function after musculoskeletal tumor reconstruction [39,40].

#### 2.3.3 Radiographic osteoarthritis

Standardized weight-bearing mortise and lateral views of the rotationplasty pseudo-knee and contralateral ankle, as well as anteroposterior and lateral views of both hips, were obtained. The full imaging and data acquisition protocol has been published previously [29]. All radiographs were independently screened for osteoarthritis (OA) using the Kellgren–Lawrence (KL) scale [41]. This assessment was performed by two specialists, with disagreements resolved by a third reviewer. Patients with a KL grade of ≥2 were classified as having radiographic OA. A previous study using the same dataset in this patient group reported excellent interobserver reliability (Cohen’s κ = 0.81; 95 % CI: 0.68–0.93) with an absolute agreement rate of 87 % [29].

#### 2.3.4 3D gait analysis

Gait analysis was assessed using a 12-camera Vicon Vantage V system (VICON, Oxford, UK), on a 12-meter walkway at a self-selected walking speed. Ground reaction forces (GRF) were recorded via two embedded force plates (OR6-7, AMTI, Watertown, USA; 1000 Hz).

Reflective markers were placed according to the Plug-in-Gait model. Participants wore their own footwear and prosthesis and used a walking aid if required. A trial was valid if both feet made full contact with the force plates, and all markers were tracked. A minimum of three valid trials were collected per participant.

#### 2.3.5 Energy cost of walking

A 6-minute walk test at a self-selected comfortable speed on a 35-meter indoor oval track was performed. Breath-by-breath gas exchanges, oxygen uptake (VO_2_) and carbon dioxide output (VCO_2_), was measured using a portable gas analysis system (K5, Cosmed, Rome, Italy). Participants used their own footwear, prosthesis, and walking aid if necessary. The K5 has been shown to be a valid and reliable device for the assessment of VO2 and VCO2 during self-paced indoor walking in healthy individuals [42,43]. Participants were instructed to refrain from eating or drinking sugary beverages for at least 90 min prior to testing.

#### 2.3.6 Data processing

Gait data was processed using standard Vicon pipelines. Sagittal joint kinematics and kinetics were time-normalized to the gait cycle (0–100 %) and averaged across valid trials using custom Matlab scripts (R2021a, The Math Works, Natick, MA, USA).

Spatiotemporal parameters that were collected were cadence, step length, double support time, and walking speed. Energy cost of walking was calculated from VO_2_ and VCO_2_ during steady state walking (minutes 4–6) and expressed as J·kg^−1^·m^−1^ [44]. Statistical Parametric Mapping (SPM) was applied to analyze full-waveform differences in joint angles, moments, powers, and GRFs across the gait cycle.

### 2.4 Statistical analysis

Statistical analyses were performed using SPSS version 28.0 (IBM Corp., Armonk, NY, USA), except for statistical parametric mapping (SPM), which was conducted in MATLAB R2023b (MathWorks, Natick, MA, USA). Normality of continuous variables was assessed using Shapiro–Wilk tests and visual inspection of distribution plots. Continuous data were presented as means with standard deviation (SD), and categorical data as frequencies with percentages.

Comparisons between minors and adults were performed for QoL, radiographic OA, patient-reported functioning, energy cost of walking, spatiotemporal gait parameters, and full-cycle gait waveform data (sagittal plane joint angles, moments, powers, and vertical and mediolateral GRFs). Continuous variables were compared using independent t-tests or Mann–Whitney U-tests as appropriate. Categorical variables were compared using Chi-square or Fisher’s exact tests depending on cell counts. Gait waveforms were analyzed using SPM-based two-tailed t-tests across the full gait cycle. Within the SPM framework, statistically significant differences were defined as waveform regions exceeding the critical threshold based on random field theory, with α = 0.05 controlling the type I error rate across multiple correlated time points [45,46].

Given the exploratory design and limited sample size, no adjustment for multiple comparisons was applied. Missing data were excluded listwise. A priori power calculation was not possible due to the absence of comparable prior studies.

### 2.5 Literature review

To provide an overview of existing literature a systematic search of PubMed, Embase and the Cochrane Library was performed on 1 July 2025 to identify all published literature on rotationplasty in adult patients. Title/abstract and mesh terms related to rotationplasty were combined with adult-specific terms (Appendix I Table 1). Two reviewers independently screened all records. Studies were included if they (1) involved patients ≥18 years at the time of surgery, (2) reported clinical or functional outcomes, and (3) were written in English, German, Dutch, or French. Articles were excluded if they reported only group-level data that included minors without providing individual patient data. Discrepancies in study selection were resolved by discussion between the two reviewers. Because nearly all publications included were case reports or very small case series, no formal quality or risk-of-bias assessment was performed, as methodological quality is inherently low. Data from the included studies were extracted and summarized in tabular form, with a narrative synthesis of functional and clinical outcomes.

## 3 Results

### 3.1 Cohort study

Of the 33 patients, 24 underwent rotationplasty as minors and nine as adults (10.7 ± 3.5 vs. 27.9 ± 8.7 years; MD 17.3, 95 % CI 13.0 to 21.5, *p* < 0.001). The mean follow-up time did not differ significantly between the two groups (33.7 ± 4.5 vs. 30.2 ± 4.4 years; MD –3.5, 95 % CI –7.1 to 0.1; *p* = 0.06). Also, there was no significant difference in sex distribution between the two groups, while mean BMI was significantly lower in the minor group (24.6 ± 3.5 vs. 27.8 ± 3.6 kg/m^2^; MD 3.2,95 % CI 0.3 to 6.1*; p = 0.03*). Osteosarcoma was the predominant diagnosis in both groups (87.5 % in minors vs. 55.6 % in adults), whereas benign indications were observed exclusively in adults (22.2 %). Complication rates and types did not differ between the groups. Social status (employment, partnership status) did not differ significantly between the groups (Table 1).

**Table 1:** Baseline characteristics of patients undergoing rotationplasty, stratified by age at surgery.

	Age at surgery				
	<18	≥18	Mean difference (95 % CI)		p-value
N	24	9			
*Mean (SD)*					
Age at intervention (yrs)	10.7 (3.5)	27.9 (8.7)	17.3 (13.0 to 21.5)		<0.001
Age at test day (yrs)	44.4 (5.4)	58.2 (8.7)	13.8 (8.7 to 18.9)		<0.001
FU-time (yrs)	33.7 (4.5)	30.2 (4.4)	−3.5 (−7.1 to 0.1)		0.06
BMI (kg/m^2^)	24.6 (3.5)	27.8 (3.6)	3.2 (0.3 to 6.1)		0.03

*N (%)*					
Female (yes)	12 (50.0)	6 (60.7)			0.39
Indication for rotationplasty Malignant* Benign^♦^	24 (100) 0 (0)	7 (77.8) 2 (22.2)			0.07
*Complications N (%)*					
Vascular disease	1 (4.2)	1 (11.1)			0.47
Neurological deficit	3 (12.5)	1 (11.1)			0.91
Non-union	0 (0)	0 (0)			N/A
Infection	4 (16.7)	0 (0)			0.55
Bone necrosis	1 (4.2)	0 (0)			1.00
Having a partner (yes)	20 (83.3)	7 (77.8)			0.71
Work (yes)	20 (83.3)	6 (60.7)			0.30

Values are presented as mean ± standard deviation (SD) or number (%). Adults were defined as patients ≥18 years at the time of surgery; minors as <r years. BMI = body mass index.*Osteosarcoma; Ewing sarcoma; malignant fibrous histiocytoma; malignant GCTB, ♦Hemangioma and proximal femoral focal deficiency. N/A = not applicable

### 3.2 Quality of life and satisfaction

Individuals who underwent rotationplasty during adulthood reported significantly higher physical health scores at follow up compared with those who underwent the procedure during childhood (SF-36 PCS 51.6 ± 5.7 vs. 45.5 ± 11.3; MD 6.1, 95 % CI 0.0 to 12.3; *p* = 0.05). However, no significant differences were observed in mental health and overall satisfaction (p < 0.13) (Table 2).

**Table 2:** Quality of life, satisfaction, and radiographic osteoarthritis outcomes in adults versus minors.

	Age at surgery				
	<18	≥18	Mean difference (95 % CI)		p-value
*PROMs, mean (SD)*					
SF-36 PCS	45.5 (11.3)	51.6 (5.7)	6.1 (0.0 to 12.3)		<0.05
SF-36 MCS	50.7 (9.9)	56.1 (5.2)	5.3 (−1.7 to 12.5)		0.13
Overall satisfaction	82.0 (27.1)	96.2 (5.4)	14.3 (−4.5 to 33.0)		0.12

*Presence of OA, N (%)*					
Pseudo-knee	9 (42.9)	4 (44.4)			1.00
Contralateral Ankle	3 (15.0)	0 (0)			0.53
Ipsilateral hip	5 (23.8)	5 (55.6)			0.12
Contralateral hip	3 (15.0)	0 (0)			0.54

SF-36 PCS = Physical Component Summary; SF-36 MCS = Mental Component Summary; VAS = Visual Analogue Scale (0–100); OA = osteoarthritis; KL = Kellgren–Lawrence scale (grade ≥2 classified as OA). Values are mean ± SD or number (%).

### 3.3 Radiographic osteoarthritis

**Fig. 1:**
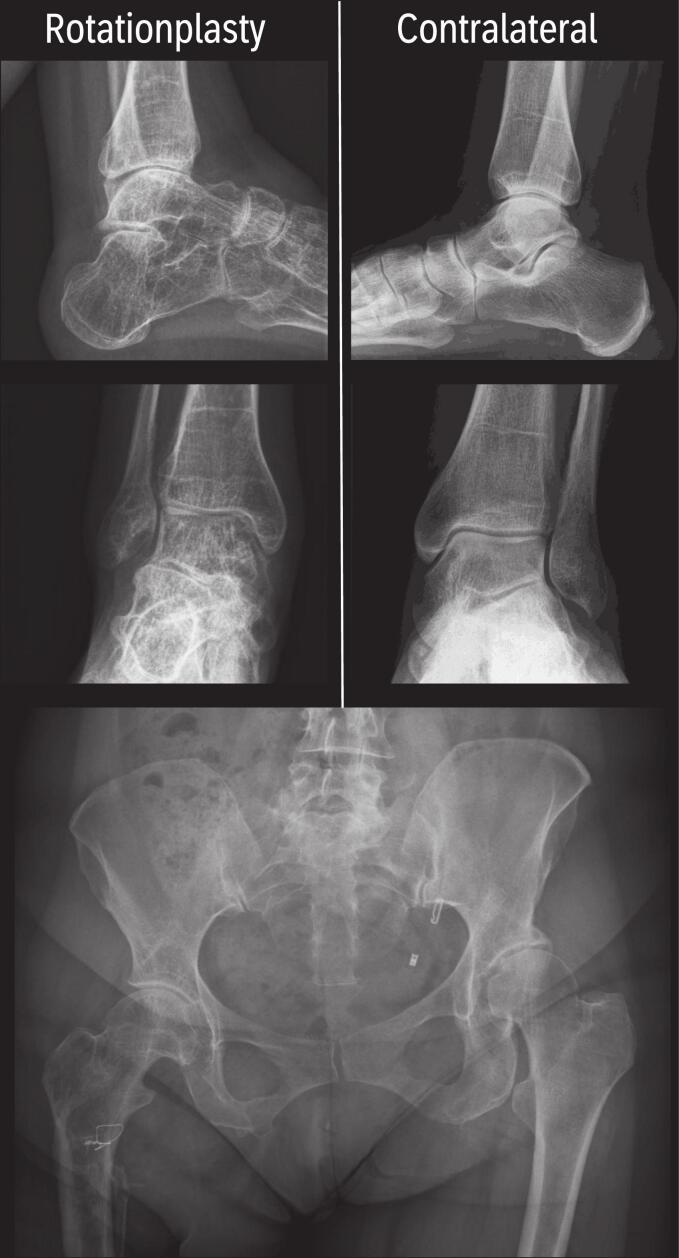
Degeneration after rotationplasty: Demo case comparing operated and contralateral leg independent of age at surgery.

No significant differences in the presence of OA were observed between the age groups in the pseudo-knee, contralateral ankle, or hips (p *>* 0.05) (Table 2), (Fig. 1).

### 3.4 Functional outcomes

No significant differences were found between the age groups in patient-reported functional outcomes (TESS: *p* = 0.14; MSTS: *p* = 0.48), energy cost of walking (*p* = 0.70), comfortable walking speed (*p* = 0.06), cadence (*p* = 0.31), or double support phase from the rotationplasty leg to the contralateral leg (*p* = 0.12). However, stride length was significantly shorter in the adult group (1.3 ± 0.1 vs. 1.5 ± 0.1 m; MD –0.2, 95 % CI –0.3 to –0.1; p < 0.01), and the double support phase from the contralateral leg to the rotationplasty leg was significantly longer (13.8 ± 2.5 % vs. 11.7 ± 1.6 % of gait cycle; MD 2.1, 95 % CI 0.1 to 4.1; p = 0.01) (Table 3).

**Table 3:** Functional outcomes and gait parameters in adults versus minors after rotationplasty.

	Age at surgery				
	<18	≥18	Mean difference (95 % CI)		p-value
*Mean (SD)*					
TESS	86.5 (11.2)	92.6 (6.7)	6.0 (−2.1 to 14.2)		0.12
MSTS	23.9 (5.8)	25.4 (4.3)	1.5 (−2.9 to 5.9)		0.48
Energy cost of walking (J/kg/m)	4.4 (0.6)	4.3 (0.7)	−0.1 (−0.6 to 0.5)		0.60
Walking speed (m/s)	1.3 (0.2)	1.2 (0.1)	−0.1 (−0.2 to 0.0)		0.06
Cadence (step/min)	99.6 (6.6)	102.4 (6.6)	2.8 (−2.7 to 8.2)		0.31
Stride length (m)	1.5 (0.1)	1.3 (0.1)	−0.2 (−0.3 to −0.1)		<0.01
Double support R to C (%GC)	12.8 (2.1)	14.0 (1.6)	1.3 (−0.4 to 2.9)		0.12
Double support C to R (%GC)	11.7 (1.6)	13.8 (2.5)	2.1 (0.1 to 4.1)		0.01

TESS = Toronto Extremity Salvage Score; MSTS = Musculoskeletal Tumor Society Score; VO_2_ = oxygen uptake; VCO_2_ = carbon dioxide output. Values are presented as mean ± SD. Significant group differences (p < 0.05) are highlighted in bold. R = rotationplasty leg; C = contralateral leg; GC= gait cycle.

### 3.5 Biomechanical analysis

**Fig. 2:**
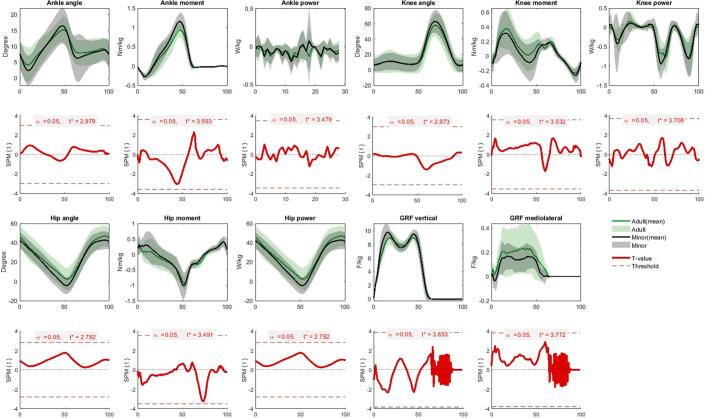
Sagittal-plane gait kinematics, kinetics, and ground reaction forces in adults versus minors. Upper rows (green/grey lines): Joint angles, joint moments, joint powers, and vertical and mediolateral ground reaction forces across the gait cycle (0–100 %) for patients with a rotationplasty in adulthood (green) versus the rotationplasty patients in childhood (grey). Curves show group means with SD’s. Lower row (red line): These panels show the statistical comparison between the two groups corresponding with the upper panel. The solid line is the Statistical Parametric Mapping (SPM) t-value over the gait cycles; the dashed red line is the threshold needed to call a difference “significant”. (For interpretation of the references to colour in this figure legend, the reader is referred to the web version of this article.)

No significant differences were observed between the age groups in sagittal plane kinematics, kinetics, or ground reaction forces (p > 0.05) (Fig. 2).

### 3.6 Literature review

The literature search retrieved 155 records after removal of duplicates, of which 22 met the inclusion criteria. In total, these studies described 51 adult patients, comprising 17 single case reports and five small case series. Reported ages ranged from 18 to 80 years, with follow-up ranging from 5 months to 31 years. Indications for rotationplasty included primary malignant tumors (n = 25, 49 %), failed limb salvage or endoprosthetic failure (often due to chronic infection or non-union) (n = 22, 43 %), trauma (n = 5, 10 %) and failed total knee arthroplasty (n = 2, 4 %). Complications were reported in 49 of 51 patients (96.1 %), with 16 patients (32.7 %) experiencing at least one event. The most common were thrombotic events and non-union (each n = 3, 6.1 %), followed by skin necrosis, ankle stiffness, and numbness (each n = 2, 4.1 %). Infection, malrotation, sepsis, and postoperative depression each occurred in 1 patient (2.0 %). Secondary amputation occurred in 4 patients (8.2 %), including one due to local recurrence, one due to stiffness of the ankle, and two due to vascular complications.

QoL was reported in only five cases, each using a different assessment tool [6,24,25,27,47]. All studies that reported patient satisfaction described good satisfaction (n = 8, 100 %) and stated that they would choose rotationplasty again[5,17,19,27,48,49]. Functional outcome scores were available for 18 patients, with MSTS scores ranging from 63 % to 100 % and TESS scores between 85 % and 95 %[5,6,19,20,22,24,27,47]. Most patients were able to ambulate independently. However, none of the studies included objective gait analysis; in two cases, gait was assessed only through visual observation, with both reports describing a pattern similar to that of above-knee amputees [21,48]. Comfortable walking speed was reported in four cases, ranging from 0.7 to 1.5 m/s [6,21,24,48]. In one case, degeneration assessed, with no changes observed 15 years postoperatively [17]. Despite occasional complications, most patients expressed overall satisfaction (Table 4).

**Table 4:** Literature overview.

	N	Age	Indication rotationplasty	Type	FU (months)	Complications	QoL	Function	Satisfaction/other
Brigman et al. 2003 [5]	1	26	Failed limb salvage due to tumor recurrence	AI	25	No		Able to walk approximately 1/2 mile with a cane, did not use a cane for household ambulation. **Flexion/extension: ROM:** 70° **MRC:** 4+/5 **MSTS:** 67 %	Satisfied with his limb, would have chosen rotationplasty again
Cahill et al. 2019 [53]	1	25	Chronic bone and soft tissue infection of the right thigh following resection andradiation of epithelioid sarcoma	BIIIb	NS	No		**ROM:** 90° of knee flexion Ambulates very comfortably without a walking aid and can hop up and down.	
Chen et al. 2023 [27]	2	47	1) Infectious nonuion femoral fracture due to nonunion after radiotherapy for hemangioendothelioma, infectious nonunion. 2) Liposarcoma in the thigh	BIIIb	1) 24 2) 24	1) No 2) Skin necrosis, healed without surgery	1**) SF-36:** 56 (mean 50) 2) **SF-36:** 52(mean 50)	1) Could walk comfortably without a walker's aid. **MSTS:** 72 % **FMA:** 53 (max 70) **TESS:** 93 % 2) Could walk comfortably without a walker's aid. **MSTS:** 63 % **FMA:** 47 (max 70) **TESS:** 88 %	1) − 2) Expressed satisfaction with her current appearance and functionality
Compston et al. 2020 [20]	1	20	Failed limb-salvage with allograft reconstruction (nonunion) after high-grade osteosarcoma of left distal femur	AI	12	NS		**TUG**: 5.3 s **MSTS**: 100 % **TESS:** 95.3 % ROM pseudo-knee: **Flexion/extension:** 100–0-10 ° **Inversion/eversion:** WNL **Flexion/extension: MRC:**5/4 **Inversion/eversion: MRC:** 4/3+	No Pain, no fatigue
Dodwad et al. 2014 [7]	1	38	Infected total knee arthoplasty	AI	6	No		Can assume a squattingposition during baseball. **Flexion/extension: ROM:** 90°	Returned tofulltime work
Dumont et al. 2010 [21]	1	62	Infected knee arthroplasty	AI	26	No	−	Could walk 800 m without crutches, could drive his car **Flexion/extension: ROM:** 80–0-10 **MRC**: 5/5 **Gait:** Both knees extended at heel-strike and no loading response on the side of the rotationplasty because of the stiffness of the prosthesis, and on the contralateral side because of the limited dorsal flexion of the foot **Walking speed:** 0.8 m/s	He and his family had no more trouble with the cosmeticappearance of the limb
Fukushima et al. 2023 [24]	1	37	Failed endoprosthetic replacement for synovial Sarcoma of the distal femur	AI	12	No	**QOL-C30:** 100 (max 100)	**TUG:** 6.25 s **MSTS:** 80 % **TESS:** 88.3 % **Muscle strength pseudo-knee:** 16.7 kg/f **Walking speed:** 0.7 m/s	Returned to work
Gaillard et al. 2021 [47]	1	38	Complex septic distal femoral non-union following allograft reconstruction after osteosarcoma	AI	6	No	**EQ5D-3L:** very satisfactory	**MSTS:** 73 % **TESS:** 87 %	Health condition VAS:90/100
Gulia et al. 2023 [22]	11	18–48	Failed limb-salvage	AI/ AII	24–204	1 vascular complication in which amputation was needed		**MSTS (10pt):** 26.5 (1.4 SD) (max 30)	
Hahn et al. 2003 [23]	15	18–37	Bone tumor around the knee	NS	6–95	Nonunion (n = 3) Malrotation (n = 1) Amputations (n = 2, 1 due to local recurrence, 1 due to stiffness of the ankle) Sepsis (n = 1)		**ROM pseudo-knee:** **1:** 0/90, **2:** 30/85, **3:** 5/90, **4:** 20/90, **5:** 20/100, **6:** 0/80, **7:** 15/75, **8:** −5/90, **9:**-5/75, **10:** 10/90, **11:** 20/80, **12:** 30/90, **13:** 10/90, **14:** 30/90, **15:** −10/70	
Hardes et al. 2008 [17]	3	1) 62 2) 70 3) 63	Soft-tissue sarcoma	AI	6–180	1.Thrombosis		Two patients needed a cane for a gait distance > 200 m. The walking distance even with support wasreduced but ranged from 500 to 2000 m. **Enneking means:** 19 (max 30) **EMG:** reduced activity of the medial gastrocnemius muscle but good adaptation of themuscles to the new function	All patients were amenable tohaving the same surgery again if necessary. In patient number 2 no signs of degenerative joint diseaseof the ankle
Hsieh et al. 2023 [60]	1	19	Traumatic amputation	AII	18	Hypersensitivity and pain (visual analog score: 5), improved with no pain after 18 months		**ROM pseudo-knee:** 20–0-70	
Kawai et al. 1995 [61]	1	19	Osteosarcoma	AI	13.3	No		Walked well without aid. **Enneking:** 87.9 % **ROM pseudo-knee:** 90	
Klos et al. [26]	1	80	Severe traumatic soft tissue injury of the thigh	AI	24	Developed reactive depression, after treatment this became stable.		Able to propel himself in his wheelchair, which was his main form of locomotion. Could also move around the house using hisprosthesis and a walking frame. **Pseudo-knee:** **ROM**: 0–30-75 **MRC**: 5/5	
Krettek et al. 1997 [48]	1	31	Traumatic fracture and infection of femur	A1	18	Limited range of motion while getting into and out of vehicles. Unsatisfying walking pattern		Could walk five kilometers without aid. **Pseudo-knee:** **ROM:**3–75 **MRC**: 5/5 **Gai:** A pattern like that of someone who has had anabove-the-knee amputation, with the knee extended at heel-strike. **Walking speed:** 1.48 m/s	Satisfied with the limb and had no pain and could return
Lu et al. 2022 [54]	1	37	Severely crushed floating knee in ablast injury	AI	48	The injured tibial nerve caused left footnumbness that prevented walking for longer than 3 h at a time.		Could ambulate independently. **ROM pseudo-knee**: 15–60	VAS pain: 2 (out of 10
Morri et al. 2017 [6]	1	31	Dedifferentiated parosteal osteosarcoma of the femur	AI	12	No	**SF-36 (per category max 100)** **Physical Functioning:** 60 **Role-Physical:** 50 **Bodily Pain:** 100 **General Health:** 76 **Vitality:** 80 **Social Functioning:** 75 **Role-Emotional:** 100 **Mental Health:** 84	**TUG:** 7.7 **MSTS:** 80 % **TESS:** 87 % **Knee ROM:** 0–95 **MRC**: 4.5/5 **Walking speed:** 1.0 m/s	
Otsuka et al. 1998 [56]	1	20	Osteosarcoma	AI	24	No		Can walk well. **pseudo-knee ROM:** −10––100	Married
Petri et al. 2012 [25]	1	18	Grade IIIC openfracture of the distal femur	AI	372	NS	**SF-36** **PCS:** 56 **MCS:** 55	Only sitting sometimes causes inconveniencies due to poor soft tissue coverage of thetibia. **Knee:** **ROM:** 10–0-70 **Strength:** 5 (max 5) **Knee flexion power:** 8.1 nm Increase of heart rate: 85 to 102/min whileincreasing walking speed from 2.0 to 2.5 km/h.	Returnedto work as a craftsman within 18 months after surgery
Salzer et al. 1981 [62]	3	1) 24 2) 20 3) 32	All Osteosarcoma	AI	1) 34 2)17 3) 6	1) − 2) Thrombotic occlusion with ischemia of the foot occurred post-operatively, with the result that the lower leg had to be amputated		**ROM pseudo-knee** 1) 0–70 2) − 3) 0–90	
Tye et al. 2021 [49]	1	26	Traumatic Gustilo-Anderson Type IIIA open distal femoral shaft fracture	AI	36	Underwent several repeat I&D procedures and a split-thickness skin graft procedure for skin coverage to his anteromedial thigh		**ROM:** 90 degrees of knee flexion Descriptive: Adequate flexion, extension, and abduction of the reconstructed limb with functional strength of his lower limb muscles.	Extremely satisfied with the results and has slowly resumed activities including dancing, swimming, and basketball
Wicart et al. 2002 [19]	1	26	Endoprosthetic failure due to infection, for tage-IIB malignant tumour of the distal femur	AI	63	No		**Pseudo-knee ROM:**0–95 **MSTS**: 28/30	Patients and families confirmed that they would choose this treatment again if the need arose. Found suitable jobs and a marital

EMG = electromyography; FMA = Functional Mobility Assessment; MCS = mental component score; MRC = Medical Research Council; MSTS = musculoskeletal society score; PCS = physical component score; ROM = range of motion; TESS = Toronto extremity salvage score; TUG = time up and go; WNL = within normal limits.

## 4 Discussion

This study provides valuable insights into the long-term outcomes of rotationplasty in individuals who had the surgery during adulthood versus those who underwent the procedure during childhood. Although rotationplasty is primarily performed in pediatric patients with malignant bone tumors, our findings suggest that adults can achieve comparable psychosocial, functional, and biomechanical outcomes. Notably, individuals who underwent rotationplasty in adulthood showed slightly higher physical QoL scores. Additionally, they exhibited a shorter stride length and a longer double support phase from the contralateral side toward the rotationplasty side. Overall, the literature indicates good QoL and functional outcomes, despite complications, although objective gait data remain absent.

Contrary to the common assumption that pediatric patients physically adapt better to rotationplasty [7,27,28], our study showed that patients who underwent rotationplasty in adulthood reported significantly higher PCS SF-36 scores. Although the mean difference was only 6 points, it exceeds the minimal clinically important difference (MCID) of 5 points reported for the SF-36 PCS in orthopedic oncology patients [50]. In the literature, only five adult cases have reported QoL outcomes, all describing satisfactory results [6,24,25,27,47]. Several factors may explain the favourable outcomes observed in adults [5,20,22,51]. Possible explanations include greater psychological preparedness, more realistic expectations, and a more rational decision-making process in adults, as the prefrontal cortex, responsible for executive functions like planning and impulse control, continues to mature into early adulthood [51]. Moreover, many adult cases involved patients with failed reconstructions and multiple prior surgeries. For these individuals, rotationplasty may have offered a definitive and stable solution after prolonged periods of infection, repeat surgery, and limited function, with relatively few complications reported [5,20,22].This patient selection may partly explain the favorable outcomes observed in adults, whereas in children rotationplasty is more often performed as a primary procedure.

Although previous studies have raised concerns about an increased risk of degenerative changes in the lower limb joints after rotationplasty [28,29,52], our findings suggest that the long-term radiographic prevalence of OA is not markedly affected by age at surgery. However, contralateral OA was observed only in the childhood group, which may reflect higher activity levels in younger patients. Only one adult case in the literature reported joint degeneration, with no radiographic changes observed 15 years after surgery at the age of 70 [17]. While follow-up durations in our study were comparable between groups, younger patients were still relatively young at the last assessment, whereas adults had already reached an age at which OA is more likely, as also reflected in the high prevalence of ipsilateral pseudo-knee and hip OA in this group. Thus, OA prevalence in the pediatric group may increase with advancing age, underlining the importance of extended follow-up to fully capture long-term differences.

The absence of significant differences in TESS, MSTS and most gait parameters indicates that similar functional outcomes can be achieved regardless of the age at surgery. These findings align with earlier reports suggesting that adults can reintegrate successfully into daily life following rotationplasty in carefully selected cases [[5], [6], [7],^20^,^25^,^27^,^47^,^53^,^54^]. Comparable TESS and MSTS scores have been reported in 18 other adult cases described in the literature [5,6,19,20,22,24,27,47,55]. Reported ranges of motion confirm that the pseudo-knee can provide adequate flexion and extension to support functional gait [6,7,19,20,23,56].

Regarding spatiotemporal gait parameters, only minor gait differences were observed, with adults showing an approximately ∼2 % longer double support phase and slightly shorter stride lengths. Earlier research examining patients 2–7 years after surgery suggests that individuals who undergo rotationplasty develop compensatory strategies to enhance stability. These adaptations include a slower walking cadence, shorter stride length, and a longer period of double support compared with healthy controls [57].. Similar adaptations, such as reduced stride length, have been documented in lower-limb amputees to improve balance [58,59]. Some studies have suggested that elderly patients may experience lower functional outcomes, potentially due to reduced physical reserves or comorbidities [17,21,26]. This could also apply to our adult cohort, given their older age at follow-up. In certain cases, concomitant (traumatic) sciatic nerve injury was present, which may further impair function [54]. In our cohort these small changes are unlikely to be clinically meaningful and may represent mild compensatory strategies to enhance stability. Nevertheless, the largely comparable gait outcomes in our cohort suggest that age at surgery alone does not substantially impair long-term results after rotationplasty. Further research is needed to clarify the influence of age-related factors and nerve involvement, particularly in older or medically complex patients.

This study has several limitations. Most notably, the small sample size, particularly in the adult rotationplasty group, limits statistical power and the findings should therefore be interpreted with caution. Nonetheless, given the rarity of rotationplasty performed in adulthood and the complete absence of prior data on long-term outcomes in this specific population, this study provides novel and clinically relevant insights into patient-reported QoL, function, gait biomechanics, joint degeneration, and energy cost of walking. Adults in our cohort had significantly higher BMI and were older at assessment, both factors that could negatively affect gait. However, such effects were not clearly observed in the data, which may suggest that adults maintain high functional levels despite these potential disadvantages. However, because these variables may act as confounders, and the study was not powered to detect small effect sizes, these findings should be interpreted with caution. Regarding the characteristics of the initial cohort, all procedures were Winkelmann type A1 resections of the distal femur or proximal tibia, performed by a single surgeon using a standardized technique, which resulted in a relatively homogeneous group and likely minimized potential procedural bias. Outcomes for adults undergoing rotationplasty for non-oncological indications, such as trauma or failed reconstructions, remain extremely particularly scarce. Given the heterogeneity and methodological limitations of the available literature, our review findings should be interpreted as exploratory rather than definitive.

Further research is required to evaluate the safety and efficacy of rotationplasty in these settings. Until more evidence is available, rotationplasty should be reserved for carefully selected patients, following multidisciplinary evaluation and shared decision-making.

## 5 Conclusion

This study demonstrates that individuals who underwent rotationplasty during adulthood can achieve comparable long-term quality of life, and functional outcomes and gait performance to those treated during childhood and even showed slightly higher physical QoL scores. Contrary to the common perception that rotationplasty is best suited for younger patients, our findings suggest that age at surgery alone does not preclude favorable outcomes. Rotationplasty could be considered a viable reconstructive option in carefully selected adults, offering long-term quality of life, high satisfaction, and durable functional outcomes despite complications.

## Funding sources

This study was supported by funding from the JKF-Fonds, MKE-Stichting and Stichting Vrienden Integrale Oncologische Zorg (VIOZ). Each author certifies that there are no funding or commercial associations (consultancies, stock ownership, equity interest, patent/licensing arrangements, etc.) that might pose a conflict of interest in connection with the submitted article related to the author or any immediate family members.

## CRediT authorship contribution statement

**Gitte G.J. Krebbekx:** Writing – review & editing, Writing – original draft, Visualization, Validation, Software, Resources, Project administration, Methodology, Investigation, Funding acquisition, Formal analysis, Data curation, Conceptualization. **N.F.J. Waterval:** Writing – review & editing, Writing – original draft, Supervision, Methodology, Investigation, Formal analysis, Data curation, Conceptualization. **M.A. Brehm:** Writing – review & editing, Writing – original draft, Supervision, Resources, Conceptualization. **M.J.C. Duivenvoorden:** Writing – review & editing, Writing – original draft, Investigation, Formal analysis, Data curation, Conceptualization. **I.N. Sierevelt:** Writing – review & editing, Supervision, Methodology, Investigation, Formal analysis, Data curation, Conceptualization. **J.A.M. Bramer:** Writing – review & editing, Validation, Supervision, Resources, Project administration, Conceptualization. **G.M.M.J. Kerkhoffs:** Writing – review & editing, Supervision, Resources, Project administration, Funding acquisition, Conceptualization. **F.G.M. Verspoor:** Writing – review & editing, Writing – original draft, Validation, Supervision, Resources, Project administration, Methodology, Investigation, Funding acquisition, Conceptualization.

## Ethics approval

The study has been approved by the medical ethical committee of the Amsterdam University Medical Centre, Amsterdam, The Netherlands. Approval number: NL72453.018.20.

## Declaration of competing interest

The authors declare that they have no known competing financial interests or personal relationships that could have appeared to influence the work reported in this paper.
