# Supplementary material for: Rotationplasty performed in adults versus minors: a comparative study of long-term quality of life, functional and biomechanical outcomes
Source: J Bone Oncol. 2025 Nov 26;55:100732. doi: 10.1016/j.jbo.2025.100732 (PMC12719068; doi:10.1016/j.jbo.2025.100732)
Supplement: Supplementary Data 1 [file mmc1.docx]

**Appendix I**

Table 1

| Search: PubMed Cochrane and Embase | ((rotationplasty[Title/Abstract] OR "Van Nes rotationplasty"[Title/Abstract] OR "rotation-plasty"[Title/Abstract] OR "rotationplasty procedure"[Title/Abstract] OR "rotationplasty surgery"[Title/Abstract] OR "Van Nes procedure"[Title/Abstract] OR "limb rotation surgery"[Title/Abstract]))  AND  (adult[MeSH Terms] OR adulthood[Title/Abstract] OR adult*[Title/Abstract] OR "18 years"[Title/Abstract] OR "≥18"[Title/Abstract] OR "older than 18"[Title/Abstract]) |
| --- | --- |
| Total studies | 155 (duplicates excluded) |
| Excluded studies | 73 (wrong population), 53 (only document group levels including minors), 2 (other languages), 5 (no outcomes reported only description of surgical technique) |
| Included studies | 22 |
